# Morphological elucidation of basal ganglia circuits contributing reward prediction

**DOI:** 10.3389/fnins.2015.00006

**Published:** 2015-02-05

**Authors:** Fumino Fujiyama, Susumu Takahashi, Fuyuki Karube

**Affiliations:** ^1^Laboratory of Neural Circuitry, Department of Systems Neuroscience, Graduate School of Brain Science, Doshisha UniversityKyoto, Japan; ^2^Core Research for Evolutional Science and Technology, Japan Science and Technology AgencyTokyo, Japan

**Keywords:** reinforcement learning, dopamine, striatum, actor-critic, striosome/matrix compartments, cortex, thalamus

## Abstract

Electrophysiological studies in monkeys have shown that dopaminergic neurons respond to the reward prediction error. In addition, striatal neurons alter their responsiveness to cortical or thalamic inputs in response to the dopamine signal, via the mechanism of dopamine-regulated synaptic plasticity. These findings have led to the hypothesis that the striatum exhibits synaptic plasticity under the influence of the reward prediction error and conduct reinforcement learning throughout the basal ganglia circuits. The reinforcement learning model is useful; however, the mechanism by which such a process emerges in the basal ganglia needs to be anatomically explained. The actor–critic model has been previously proposed and extended by the existence of role sharing within the striatum, focusing on the striosome/matrix compartments. However, this hypothesis has been difficult to confirm morphologically, partly because of the complex structure of the striosome/matrix compartments. Here, we review recent morphological studies that elucidate the input/output organization of the striatal compartments.

## Introduction

Reinforcement learning mechanisms have been recently proposed to be based in circuits of the basal ganglia, assuming that the dopamine nigrostriatal projection acts as a reinforcement signal pathway (Sutton, 1988; Schultz et al., 1997, 1998; Sutton and Barto, 1995; Bayer and Glimcher, 2005; Cohen et al., 2012; Hart et al., 2014). Further, Reynolds et al. (2001) have reported that synaptic potentiation in striatal neurons receiving dopamine projections depends on the input from the cerebral cortex and on the dopaminergic input from the substantia nigra. These reports have led to the hypothesis that synaptic plasticity in the striatum is under the influence of the reward prediction error, and that the striatum conducts reinforcement learning throughout the basal ganglia circuits (Barto, 1995; Montague et al., 1996; Doya, 1999, 2000a,b; Crittenden and Graybiel, 2011; Takahashi et al., 2011).

In the actor–critic models, the actor chooses actions according to some policy of behavior, and the critic offers immediate feedback that notifies the actor whether the selected action was good or bad for obtaining rewards in the long run (Barto et al., 1983; for review, see Takahashi et al., 2011). Houk et al. (1995) proposed the existence of role sharing within the striatum in reinforcement learning, focusing on the striosome/matrix compartments. According to the model, the matrix performs action selection through the basal ganglia output nuclei [the internal segment of globus pallidus (GPi)/substantia nigra pars reticulata (SNr)] (actor), whereas the striosomes perform reward prediction (critic). The projection that targets dopaminergic neurons calculates the reward prediction errors, and the actor–critic learning is processed by the dopaminergic projections to the striatum.

However, this hypothesis has been difficult to test, partly because of the complex structure of the striosome/matrix compartments in the striatum. In particular, because this structure is highly irregular and cannot be visualized without processing, such as immunostaining, identification of the exact input and output pathways is difficult. We recently elucidated the input/output organization of the striosome/matrix structure using single neuron tracing by a viral tracer with a membrane translocation signal and immunohistochemistry for vesicular glutamate transporters. In this manuscript, we review recent progress in understanding the anatomical basis of basal ganglia networks in terms of reinforcement learning models, particularly the actor-critic model.

## Striatal mosaic organization and actor–critic model

### Striosome/matrix compartments

Neurons in the striatum, unlike those in the cerebral cortex and cerebellum, do not form a layered or columnar structure. Although they appear to be randomly distributed, they are actually scattered in two embryologically different compartments called striosomes (often referred to as “patches” in rodents) and the matrix. The striosome compartment is embryologically older and the “dopamine island,” observed only during development, corresponds to patch/striosome. The matrix develops later and eventually accounts for approximately 85% of the entire striatum (Johnston et al., 1990; Nakamura et al., 2009). The matrix compartment is densely stained with acetylcholinesterase, and calbindin and somatostatin are expressed at relatively high levels (Graybiel and Ragsdale, 1978; Gerfen and Young, 1988; Gerfen, 1992). The striosome compartment is rich in μ-opioid receptors (Delfs et al., 1994; Mansour et al., 1994, 1995; Minami et al., 1994; Arvidsson et al., 1995; Kaneko et al., 1995; Ding et al., 1996; Nakamura et al., 2009).

### Cortical input and striosome/matrix structure

The striatum receives glutamatergic inputs from the cerebral cortex and thalamus (Smith and Bolam, 1990), dopaminergic inputs from the substantia nigra pars compacta (SNc), and serotonergic and noradrenergic inputs from the raphe and locus coeruleus of the brain stem. The cortical input arises from almost all areas of the cerebral cortex with a local responsiveness. For example, sensory and motor cortical areas project to the part of the putamen posterior to the anterior commissure, whereas inputs from the frontal–parietal–temporal association area project to the putamen anterior from the anterior commissure and the major part of the caudate nucleus. The limbic cortex projects to the caudate nucleus, to the anterior ventral part of the putamen, and to the nucleus accumbens (Albin et al., 1989; Alexander and Crutcher, 1990).

The cortical input to the striosomes primarily arises from the limbic cortex, specifically from the orbitofrontal cortex and insula. However, it is generally considered that the input to the matrix in the rat (Gerfen, 1984, 1989; Donoghue and Herkenham, 1986), cat (Malach and Graybiel, 1986; Ragsdale and Graybiel, 1991), and monkey (Flaherty and Graybiel, 1994) arises from a wide area of the neocortex, including the motor cortex, somatosensory area, and parietal lobe. A more distinct characteristic is the layer structure of the cerebral cortex. In rats, it has been reported that cortical layers III and Va project to the matrix and layers Vb and VI project to the striosomes and that subregions of the striatum, having both striosome and matrix compartments, are innervated by the related cortical regions (Kincaid and Wilson, 1996). Thus, the striosome and matrix compartments receive the “specific” but “related” information from the cortex to contribute to their putative roles as “actor” and “critic,” respectively. Striatal interneurons with dendrites that cross compartmental borders may be a key for sorting and integrating the corticostriatal projections.

### Thalamic input and striosome/matrix structure

Projections from the thalamus primarily arise from the intralaminar nuclei (particularly the centromedian and parafascicular nucleus), from motor relay nuclei (the anterior ventral and ventral lateral nucleus), and from the posterior thalamus (the posterior lateral nucleus and pulvinar). The role of thalamostriatal projections in learning, particularly in the learning and memory of movement has been recently verified (Kimura et al., 2004; Kato et al., 2011).

**Figure 1:**
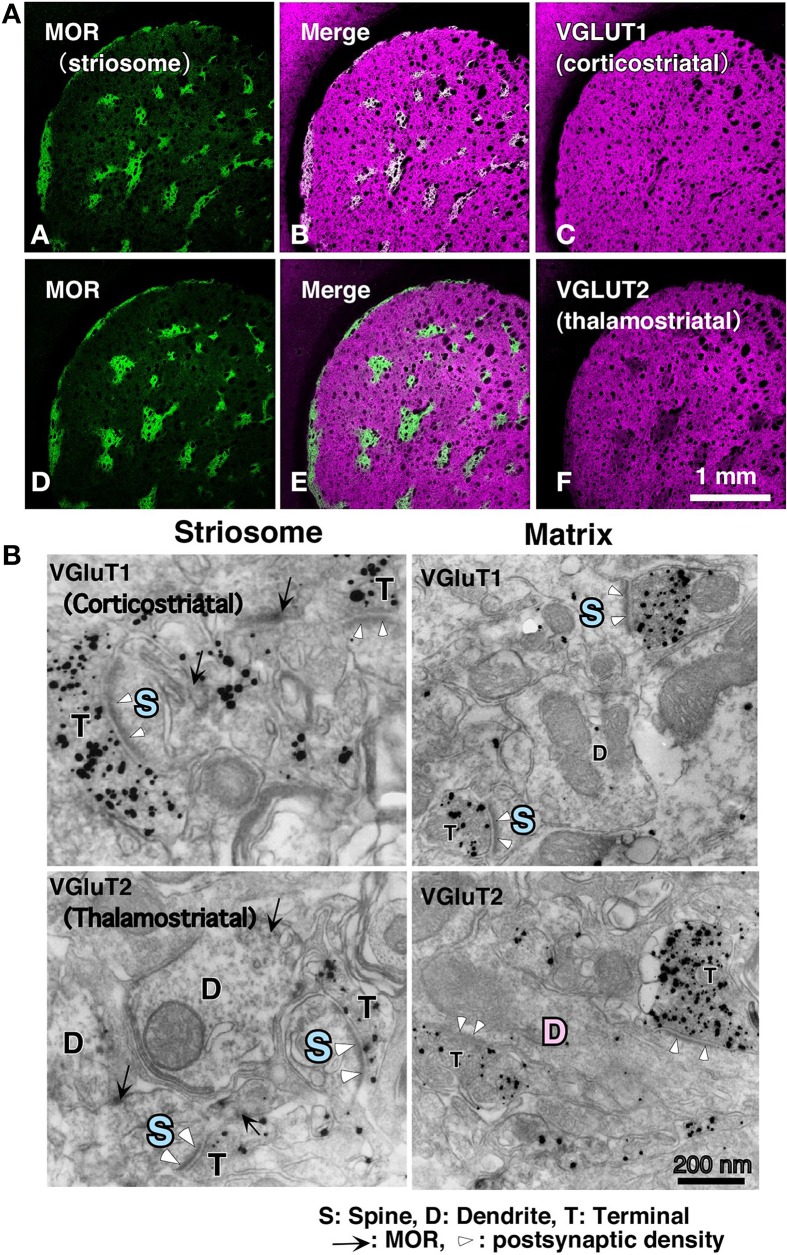
**Cortical–thalamic input and striosome–matrix structure (from Fujiyama et al., 2006 with modifications)**. **(A)** Double immunofluorescence images for MOR and either VGluT1 or VGluT2 in the neostriatum. Intense MOR-positive patch compartment corresponded to areas that were weak in VGluT2 immunoreactivity (D–F). In contrast, VGluT1 immunoreactivity distributed evenly in the neostriatum (A–C). **(B)** Immunoelectron micrographs for MOR and either VGluT1 or VGluT2 in the neostriatum. MOR, mu-opioid receptor; VGLUT, vesicular glutamate transporter.

With respect to the striatal mosaic organization, we have reported that there is approximately three times as much thalamic input to the matrix as to the striosomes (Figure 1; Fujiyama et al., 2006), using immunohistochemistry of vesicular glutamate transporters (Fujiyama et al., 2001, 2004; Kaneko and Fujiyama, 2002; Kaneko et al., 2002). The intralaminar thalamic nuclei are thought to be the major source of thalamostriatal terminals. Axons from the caudal part of the intralaminar nuclei chiefly project to the matrix compartment in monkeys (Sadikot et al., 1990, 1992), cats (Beckstead, 1984; Ragsdale and Graybiel, 1991), and rats (Herkenham and Pert, 1981; Gerfen, 1984, 1985, 1989, 2004; Deschênes et al., 1996). Conversely, the midline thalamic nuclei, including the paraventricular and rhomboid nuclei, mainly project to the neostriatal striosome compartment and ventral striatum in cat (Ragsdale and Graybiel, 1991). These results indicate that the striosome and matrix compartments not only receive different densities of thalamic inputs but also admit inputs from different thalamic nuclei. The midline nuclei receive inputs from limbic regions (Cornwall and Phillipson, 1988). Limbic afferents through the midline nuclei as well as those from limbic cortical areas are used in striosome compartment to calculate the “state value,” which is possibly a key variable in the process of reinforcement learning (for review, see Doya, 2000a,b; Doya et al., 2002). Thus, the distinct networks for the matrix and striosome compartments involve thalamic and cortical afferents.

Differences exist in the synaptic organization of thalamostriatal neurons between the striosome and matrix compartments (Fujiyama et al., 2006; Raju et al., 2006). Our quantitative analysis of ultrastructural images revealed that in striosomes, 84% of thalamostriatal synapses were made on dendritic spines, whereas in the matrix compartment, 70% were made on dendritic shafts. Contrastingly, corticostriatal terminals preferentially synapsed onto dendritic spines (~80%) in both compartments (Figure 1; Fujiyama et al., 2006). Furthermore, thalamostriatal axospinous synapses in striosomes were larger than corticostriatal axospinous synapses in either compartments (Fujiyama et al., 2006). Excitatory axospinous synapses, including corticostriatal axospinous synapses, often display a high degree of synaptic plasticity (Calabresi et al., 2000). Moreover, dendritic spines are known to rapidly and frequently change their form, presumably reflecting their plastic characteristics (Yuste and Bonhoeffer, 2001). These findings suggest that the thalamostriatal synapses on dendritic shafts in the matrix, contributing to the “Actor,” are less plastic than those on dendritic spines in the striosome compartment, contributing the “Critic” and also less plastic than corticostriatal axospinous synapses.

### Nigrostriatal dopaminergic input

Beside the excitatory glutamatergic inputs from the cortex and thalamus to the striatum, there are other important striatopetal projections, such as dopaminergic inputs from the SNc. The dopaminergic neurons in the midbrain are known to respond to the reward prediction error (Schultz et al., 1993, 1997, 1998; Schultz, 2007a,b). As described above, dopamine likely modulates synaptic plasticity between the corticostriatal afferents and striatal projection neurons (Calabresi et al., 2000, 2007; Reynolds et al., 2001; Surmeier et al., 2007; Shen et al., 2008).

Motivational value and motivational salience signals of dopaminergic neurons are distributed in an anatomical gradient across the substantia nigra and ventral tegmental area (VTA) (Bromberg-Martin et al., 2010). Anatomically, dopaminergic neurons in SNc are divided into the calbindin-positive dorsal tier and calbindin-negative ventral tier. Conventionally, the nigrostriatal projection in the rat brain has been reported to have the organization such that dopaminergic neurons in dorsal SNc chiefly project to the matrix, whereas those in ventral SNc mainly project to the striosomes (Gerfen et al., 1987). A similar segregation of nigrostriatal projections to striosomes and matrix compartments has been reported in cats and primates (Jimenez-Castellanos and Graybiel, 1987; Langer and Graybiel, 1989). However, this segregated organization was only partly supported by the results from our single neuron tracing study. We found that all single dopaminergic neurons innervated both striosome and matrix compartments, although projections from dorsal SNc neurons favored the matrix compartment and those from ventral SNc neurons favored the striosome compartment (Matsuda et al., 2009). Single dopaminergic neurons in the dorsal and ventral SNc innervated both striosome and matrix compartments is important, suggesting that identical temporal difference (TD) signals are simultaneously sent to a large number of striosome and matrix neurons.

However, how dopaminergic nigrostriatal projection processes specific reward-related learning remains unknown. One possibility is that phasically released dopamine modifies excitatory synapses. Its principal action will thus be at those cortical and thalamic synapses that are “active,” aiding the “selection” of striatal neurons to be fired (see Bolam et al., 2006; Arbuthnott and Wickens, 2007).

## How do basal ganglia mediate motor and learning?

### New aspects of direct/indirect pathways

Projection neurons in the striatum are classified into two groups, depending on their neurochemical properties and projection targets, which in turn transmit information via different routes to output nuclei, such as GPi and SNr. It is believed that the first projection group corresponds to a direct pathway, wherein the neurons containing both GABA and substance P directly project to the output nuclei, whereas the second one involves an indirect pathway, wherein the neurons containing GABA and enkephalin project to the output nuclei via the external segment of globus pallidus (GPe) and subthalamic nucleus (Albin et al., 1989; Alexander and Crutcher, 1990; Graybiel, 1990). Because these output nuclei contain GABAergic inhibitory neurons that discharge at a high rate, the projection targets in the thalamus and superior colliculus are usually in an inhibited state. Striatal projection neurons are GABAergic; therefore, excitation of these neurons by cortical inputs may lead to temporary inhibition of the output nuclei via the direct pathway and to disinhibition of the target regions (the thalamus and cerebral cortex), allowing selected movements to occur (Nambu et al., 2002). However, when the indirect pathway is activated, the target regions are further inhibited because projection neurons from GPe to the subthalamic nucleus are also GABAergic and those from the subthalamic nucleus to the output nuclei are glutamatergic. Therefore, while the direct pathway allows the expression of required movement via disinhibition during the necessary time period, the indirect pathway may be suppressing unnecessary movement and thus highlighting the outcome from the direct pathway. These findings suggest a “center–surround” model of basal ganglia function, comprising focused selection of an appropriate motor program and inhibition of competing motor programs (Mink and Thach, 1993; Mink, 1996; Hikosaka et al., 2000; Nambu et al., 2002).

Dopaminergic projections from SNc produce excitatory modulation of direct pathway neurons by an action at dopamine D1 receptors (D1Rs) and inhibitory modulation of indirect pathway neurons by an action at dopamine D2 receptors (D2Rs), effectively eliciting opposite effects in the direct and indirect pathways (Hong and Costa, 1978; Hong et al., 1978; Gerfen et al., 1990). This conceptualization has been widely accepted because it can explain clinical findings and therapeutic effects in disorders, such as Parkinson's disease.

However, it has been reported that a majority of striatal neurons are activated during movement (DeLong, 1990; Costa et al., 2004) and that both pathways are co-activated during movement initiation (Cui et al., 2013; Isomura et al., 2013). Further, recent optogenetic studies showed that both pathways were concomitantly active during sequence initiation but behaved differently during sequence performance (Jin et al., 2014). Single neuron tracing studies have revealed that almost all direct pathway neurons projected to GPe, a relay nucleus of the indirect pathway (Kawaguchi et al., 1990; Lévesque and Parent, 2005; Fujiyama et al., 2011), indicating that direct pathway neurons drive both direct and indirect pathways. Other projection systems have also been reported, such as the hyperdirect pathway (Nambu et al., 2002), cortico-dopaminergic projections (Watabe-Uchida et al., 2012), and differential cortical innervation of D1R- and D2R-positive striatal neurons (Wall et al., 2013). Furthermore, Kravitz et al. (2012) reported that positive reinforcement caused by direct pathway stimulation persists for long durations in mice, whereas punishment caused by indirect pathway stimulation was transient (Kravitz and Kreitzer, 2012; Kravitz et al., 2012). Jin et al. (2014) also showed that the basal ganglia contribute to behavior during learning rather than simple motor control (for review, see Friend and Kravitz, 2014). Thus, the original conceptualization of the direct and indirect pathways is likely to be modified through further studies, particularly those using behavioral experiments.

### Direct/indirect pathways and striosome/matrix structure

**Figure 2:**
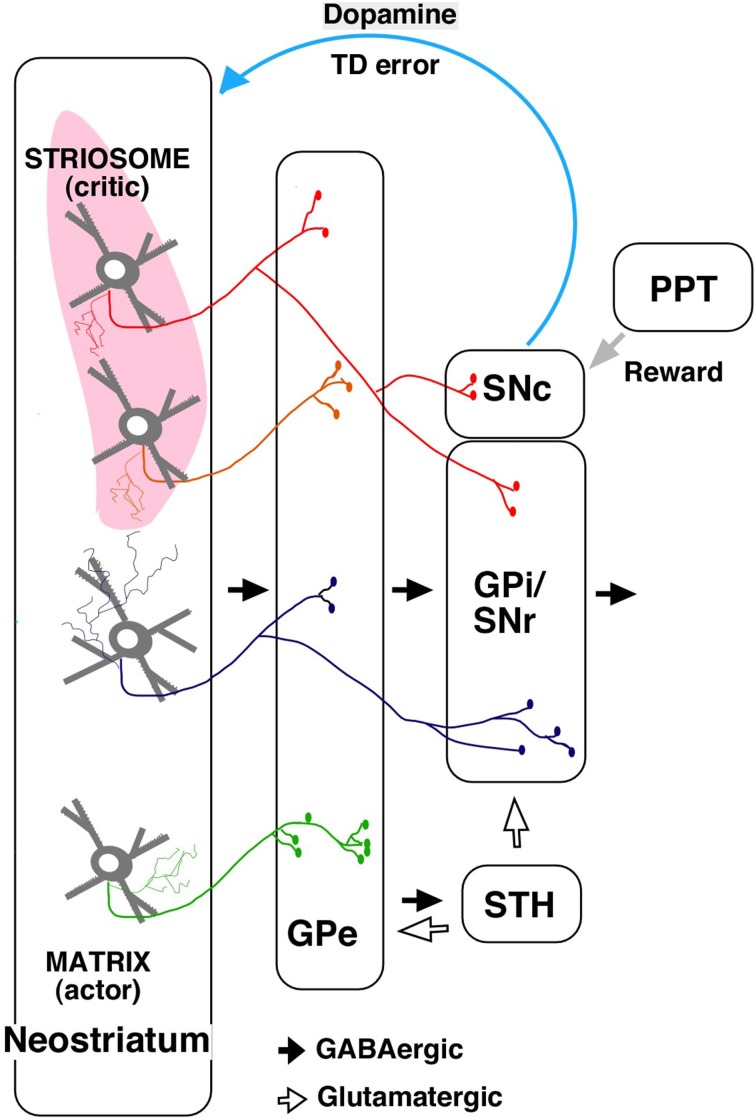
**A simplified diagram of output from the striosome–matrix structure (from Fujiyama et al., 2011 with modifications) GPe, external segment of globus pallidus; GPi, internal segment of globus pallidus; PPT, pedunculopontine nucleus; SNc, substantia nigra pars compacta; SNr, substantia nigra pars reticulata; STH, subthalamus**.

The output from striosome/matrix compartments has been difficult to examine with anterograde tracers because of the irregularities in the striatal structure. Using a single neuron tracing technique, we found that striosomes also include indirect pathway neurons projecting to GPe (Figure 2; Fujiyama et al., 2011). Further, unlike the matrix, direct pathway neurons in striosomes project not only to GPi/SNr but also directly to SNc, where dopaminergic neurons are present (Figure 2; Gerfen, 1984; Lévesque and Parent, 2005; Fujiyama et al., 2011; Watabe-Uchida et al., 2012).

The actor–critic model was supported by the finding that neurons responding to action and state values were distributed in the neostriatum (Samejima et al., 2005; Kawato and Samejima, 2007; Lau and Glimcher, 2008; Wang et al., 2013). Furthermore, dopamine signals contain information about reward and state value (Schultz et al., 1998; Schultz, 2007a,b); striosomal neurons, which directly control dopaminergic neurons in SNc, may provide the dopaminergic neurons with state-based signals. Dopaminergic neurons receive monosynaptic inhibitory input from SNr and polysynaptic disinihibitory input from the GPe (Grofova et al., 1982; Saitoh et al., 2004; Tepper and Lee, 2007; Brazhnik et al., 2008), and SNr and GPe are innervated by striosomal neurons. Hence, dopaminergic neurons may receive disinhibitory input through the SNr, and polysynaptic inhibitory input through the GPe, subthalamic nucleus, and SNr, from the striosomes. Dopaminergic neurons are also considered to receive a stochastic reward signal from the pedunculopontine tegmental nucleus and other structures (Figure 2; Okada et al., 2009). Our recent study revealed that the axonal arbors of most dopamine neurons covered a single large oval volume, occupying at most 5.7% of the total neostriatal volume. Furthermore, all single dopamine neurons we traced innervated striosomes and matrix compartments with or without volume transmission (Matsuda et al., 2009). Dopamine signals may thereby change the response properties of striatal neurons (Calabresi et al., 2000; Reynolds et al., 2001; Surmeier et al., 2007; Shen et al., 2008). The striosomal control of the dopaminergic neurons responsible for reward prediction errors is particularly relevant to its potential role as “critic;” it may serve this function by calculating the state value and sending it to dopaminergic neurons (Barto, 1995; Houk et al., 1995).

### Ventral striatum and limbic loop

In the basal ganglia, VTA–nucleus accumbens dopaminergic projection system has been considered along with the nigrostriatal dopaminergic projection system (see Groenewegen et al., 1999; Zahm, 1999). The ventral part of the striatum centered on the nucleus accumbens is called the limbic or ventral striatum; this structure is divided into a central portion (core) and periphery (shell) (Herkenham et al., 1984). GABAergic output from the shell projects either directly or through the ventromedial part of the ventral pallidum to the dorsomedial thalamic nucleus, the lateral hypothalamus, VTA, dopaminergic neurons in the substantia nigra, and is part of the mesolimbic dopaminergic projection system. The core involves a system that projects via the dorsolateral portion of the ventral pallidum to the subthalamic nucleus, SNr, and GPi, and subsequently enters the motor loop (see Groenewegen et al., 1999; Zahm, 1999). Therefore, it may help switch the input from the emotion system to the movement system. Dopamine projections from VTA to the nucleus accumbens are involved in selecting the environmental contexts leading to reward (O'Doherty et al., 2004; Canales, 2005; Goto and Grace, 2008; Humphries and Prescott, 2010; Glimcher, 2011; Morita et al., 2012, 2013; Hart et al., 2014). Takahashi et al. (2008); Takahashi et al. (2011) reported that information about task structure is represented in the orbitofrontal cortex and that it influences the computation of reward prediction error in VTA dopaminergic neurons. This presumably occurs via the ventral striatum, where the state value would be computed. However, a recent optogenetic study reported that the striatal medium spiny neurons, including striosomal neurons, synapse onto dopamine neurons only very weakly and instead strongly synapse onto GABAergic neurons in VTA (Chuhma et al., 2011), which in turn project to cholinergic neurons in the accumbens (Brown et al., 2012). Furthermore, up to 17% of accumbens shell neurons co-express D1R and D2R (Rashid et al., 2006; Bertran-Gonzalez et al., 2008; Ng et al., 2010); therefore, ventral striatal pathways are not well adapted to the direct/indirect schema of the dorsal striatum. Hence, further anatomical and physiological studies are necessary to test the synaptic connections of VTA–nucleus accumbens dopaminergic projection system.

## Conclusion

Although both involve the striatum, the direct/indirect pathways and striosome/matrix compartments have been investigated separately. Two functions of the basal ganglia— motor control and reinforcement learning—have also been traditionally discussed and understood separately. However, recent studies have shown that direct pathway neurons mediate movement, reinforcement, and reward, whereas indirect pathway neurons inhibit movement and mediate punishment and aversion (see Kravitz and Kreitzer, 2012). Recent morphological studies have shown how the striatal mosaic organization and direct/indirect pathway neurons coexist in the striatum. This suggests that striosomes are involved in controlling dopaminergic neurons responsible for reward prediction errors and for direct and indirect pathways and is of a particular relevance to both motor control and reinforcement learning. The dual anatomical and physiological pathways in the striatum may hold the key to why the basal ganglia have two functions. Further studies should examine pathway- and compartment-specific activity simultaneously in different contexts to clarify how the structure of the basal ganglia contributes to behavioral learning.

### Conflict of interest statement

The authors declare that the research was conducted in the absence of any commercial or financial relationships that could be construed as a potential conflict of interest.
